# Symptom Clusters in Cancer Patients With Bone Metastases: Subanalysis of Patients Reporting Exclusively Non-zero ESAS Scores

**DOI:** 10.4021/wjon386w

**Published:** 2011-12-19

**Authors:** Gemma Cramarossa, Emily Chen, Luluel Khan, Liying Zhang, Janet Nguyen, May Tsao, Cyril Danjoux, Elizabeth Barnes, Arjun Sahgal, Lori Holden, Flo Jon, Kristopher Dennis, Edward Chow

**Affiliations:** a Rapid Response Radiotherapy Program, Odette Cancer Centre, Sunnybrook Health Sciences Centre, University of Toronto, Canada

**Keywords:** Symptom cluster, Bone metastases, Edmonton symptom assessment system, Palliative radiotherapy

## Abstract

**Background:**

To identify symptom clusters in a subgroup of patients reporting exclusively non-zero Edmonton Symptom Assessment System (ESAS) scores at baseline, and to compare clusters with those identified in the total patient population. Secondary objective was to determine whether symptom clusters in patients with bone metastases vary when extracted using different statistical methods.

**Methods:**

An existing dataset compiled from bone metastases patients was used to identify a “non-zero” subgroup of patients reporting severity scores > 0 for all nine ESAS symptoms at baseline. Principal Component Analysis (PCA), Hierarchical Cluster Analysis (HCA) and Exploratory Factor Analysis (EFA) were performed on the non-zero subgroup to derive symptom clusters at baseline and 1, 2, 4, 8 and 12 weeks following radiation treatment. Symptom clusters in the total patient sample at baseline were also derived using the three statistical methods.

**Results:**

At baseline, different symptom clusters were identified in the non-zero subgroup compared with the total patient population regardless of the statistical method utilized. When comparing clusters derived using different statistical methods, symptom cluster results varied depending on the method employed, with a few exceptions where analogous clusters were derived using two different statistical methods at a specific time point. A complete consensus between all three methods was never observed. Only two ESAS symptoms, anxiety and depression, consistently occurred in the same cluster across different methods and over time.

**Conclusion:**

Compiling data from all eligible consenting patients may not provide an accurate overview of clustering among all the symptoms included in the assessment tool. The quantity and composition of symptom clusters identified varied based on whether patients with zero symptom severity scores were included at baseline and which statistical method was utilized.

## Introduction

Bone metastases commonly occur in advanced cancer patients. Approximately 65-75% of prostate and breast cancer patients as well as 30-40% of lung cancer patients develop bone metastases [1]. Complications can include pain, spinal cord compression, hypercalcemia, and fractures. Patients with bone metastases often experience concurrent symptoms that simultaneously influence their quality of life in a multiplicative manner [2]. As a result, symptom experience research has shifted from a traditional single symptom approach to a more inclusive method that focuses on symptom clusters [3]. A symptom cluster is described as two or more interrelated symptoms that present simultaneously [4, 5].

There is currently an abundance of research in oncology in both identifying and verifying the presence of symptom clusters [3]. Clusters can be derived either through clinical or statistical means. Thus far, the majority of studies involving the identification of symptom clusters have included all eligible consenting patients from which complete assessments are obtained. However, the inclusion of patients who did not experience all of the symptoms measured by the assessment tool, as indicated by severity ratings of zero, may interfere with accurate symptom cluster identification. Such patients, whose symptom experience lacks one or several symptoms on the assessment tool, are not representative of the entire range of symptoms. This is very significant when using brief questionnaires as symptom data collection tools.

Another concern in symptom cluster research is the use of several different statistical methods which may contribute to inconsistencies of cluster results between studies. The primary objective of this study was to examine whether symptom cluster findings differ in the subgroup of patients who reported only non-zero Edmonton Symptom Assessment System (ESAS) scores at baseline. A secondary objective was to investigate whether symptom clusters vary when examined using three commonly employed analytical methods.

## Patients and Methods

The present study analyzed the same dataset as our previous study to avoid inconsistencies due to different assessment tools or varied sample populations. Our previous study derived symptom clusters in 518 bone metastases patients receiving palliative radiation treatment using Principal Component Analysis (PCA) at baseline [6]. ESAS questionnaires were administered as part of routine clinical assessment at baseline and at 1, 2, 4, 8 and 12 weeks following radiation treatment. The ESAS is an 11-point scale that assesses the following nine symptoms: pain, fatigue, nausea, depression, anxiety, drowsiness, appetite, sense of well-being, and shortness of breath [7]. A score of zero indicates the absence of symptom and a score of ten indicates the strongest presence of a symptom. This questionnaire has been corroborated in cancer patients [8, 9]. Patient demographics, cancer history, analgesic consumption, and disease status were also recorded. All primary assessments were completed before radiation treatment. Demographics included age, gender, inpatient or outpatient status, weight loss of greater than 10% over the previous six months and Karnofsky Performance Status (KPS). Questionnaires and data were collected by a trained research assistant in person at baseline and via telephone interview at all follow-ups. Ethics approval was obtained from Sunnybrook Health Sciences Centre.

### Statistical Analysis

Principal Component Analysis (PCA), Exploratory Factor Analysis (EFA) and Hierarchical Cluster Analysis (HCA) were employed in the present study to identify symptom clusters. PCA with varimax rotation was performed on the nine ESAS items to observe any interrelationships between symptoms at each follow-up time point. This analytical method identifies which variables (symptoms) correlate with each other in a distinct pattern and groups them together forming a “component” (cluster) [10]. The assignment of symptoms to a cluster was predicted by the highest factor loading score. A significant cluster was defined as one with an eigenvalue higher than 0.8 and explaining almost 10% of the total variance. Cronbach’s alpha was calculated to determine the internal consistency and reliability of the clusters.

EFA is the most frequently used analytical method in oncology symptom cluster research. EFA is unique because it assumes symptoms in a cluster are correlated by latent factors, which bind the symptoms together [11]. The factors (clusters) with eigenvalue greater than 0.8 were retained, indicating that approximately 10% of variance in the symptom is shared with the latent factor after controlling for the correlation between factors. The maximum likelihood method followed by the varimax orthogonal rotation method was then applied to approximately multivariate normal data to assess covariance between symptoms. Together these two methods identify and finalize the items (symptoms) that belong to each cluster. The PROC FACTOR procedure in Statistical Analysis Software (SAS version 9.2) was conducted for this analysis. Cronbach’s alpha was calculated to measure the internal consistency of the derived clusters.

HCA identified clusters using average linkage between groups. This method is centered on classification, grouping similar entities together and separating this cluster from other clusters [12]. HCA is also commonly utilized to categorize groups of individuals with similar symptom profiles. Clusters were formed according to the distance between symptoms severities, determined using Euclidian distances. The PROC VARCLUS runs clusters on the basis of centroid components. The R2 values of each symptom with its own cluster and with its nearest cluster were calculated. The 1-R2 ratio is the ratio of one minus the value in the “Own Cluster” column to one minus the value in the “Next Closest” column. The lower the ratio, the more separated the clusters are. HCA produces a visual diagram, or dendrogram, of clusters identified. Symptoms that converge earlier on the dendrogram are more closely related than those that converge later.

### Subgroup analysis of patients with non-zero baseline ESAS Scores

Only patients who reported severity scores > 0 for all nine ESAS items at baseline were included in the “non-zero” subgroup. This resulted in a sample population of 129 patients. The three statistical methods were used to extract symptom clusters in this subgroup. Clusters extracted in the non-zero subgroup were compared with those derived in the original sample at baseline. PCA, EFA and HCA were then applied on follow-up data to observe the temporal pattern of symptom clusters in the “non-zero” subgroup.

## Results

Symptom clusters were observed in both patient samples except in the total patient population at 8 weeks using EFA. Symptom clusters determined using PCA, EFA and HCA at baseline in the total bone metastases patient sample were comparable. Two to three clusters consisting of two to seven symptoms each were identified depending on the statistical method used. In the non-zero subgroup, clusters identified with the PCA and EFA methods at baseline and weeks 1, 2, 4, 8 and 12 were highly diversified. Two to three clusters composed of two to seven symptoms were extracted at varying time points using these two statistical methods. The symptom clusters derived from the total patient sample and non-zero subgroup at baseline are summarized in Table 1. Appendices (www.wjon.org) depict symptom clusters derived at each follow-up for both patient groups.

**Table 1:** Baseline Symptom Clusters in the Total Patient Sample (T) and Non-zero Subgroup (NZ)

Symptom	PCA		EFA		HCA	
	T	NZ	T	NZ	T	NZ
Depression	Δ	Δ	Δ	Δ	Δ	Δ
Anxiety	Δ	Δ	Δ	Δ	Δ	Δ
Fatigue	Ο	Ο	Ο	O	Ο	Ο
Drowsiness	Ο	Ο	Ο	O	Ο	Ο
Pain	O	—	Ο	O	O	—
Nausea	X	Δ	Ο	Δ	X	Δ
Poor appetite	X	Δ	Ο	O	X	O
Dyspnea	X	Ο	Ο	O	X	Ο
Poor well-being	O	O	Ο	O	O	O

PCA: Principal Component Analysis; EFA: Exploratory Factor Analysis; HCA: Hierarchical Cluster Analysis.

Symptoms with corresponding symbols indicate they were in the same cluster.

Dash indicates the symptom was not present in any clusters.

### Baseline symptom clusters in the total patient sample versus non-zero subgroup

In our previous study, we performed PCA with varimax rotation on the nine ESAS symptoms in all 518 patients [6]. At baseline, three symptom clusters were identified. Cluster 1 consisted of fatigue, drowsiness, pain and poor well-being. Cluster 2 consisted of depression and anxiety, and Cluster 3 included nausea, appetite and shortness of breath. In the non-zero subgroup, PCA only identified two clusters that collectively account for 59% of the variance (Table 2). These clusters varied significantly from the clusters derived using the total patient sample. The first cluster composed of depression, anxiety, nausea and appetite accounted for 47% of the total variance. The second cluster, consisting of fatigue, drowsiness, shortness of breath and well-being accounted for 12% of the total variance. The symptom “pain” was not found in either of the clusters. The Cronbach’s alpha for the first and second cluster are 0.79 and 0.80 respectively.

**Table 2:** Factor Loadings and Final Communality From the Principal Component Analysis of ESAS Symptoms in Non-zero Subgroup at Baseline (n = 129)

Symptom	Component 1	Component 2	Component 3	Final communality
Depression	0.84	0.21	0.20	0.79
Nausea	0.75	0.23	-0.12	0.63
Anxiety	0.75	0.26	0.31	0.73
Poor appetite	0.54	0.40	-0.21	0.50
Drowsiness	0.12	0.89	0.06	0.80
Fatigue	0.32	0.73	0.31	0.72
Shortness of breath	0.36	0.63	-0.10	0.54
Sense of well-being	0.50	0.56	0.11	0.58
Pain	0.06	0.07	0.91	0.84
% of variance	47	12	10	
Cronbach’s alpha	0.79	0.80	—	
Eigenvalue	4.22	1.07	—	

Symptoms with bolded factor loadings were in the same cluster.

**Figure 1:**
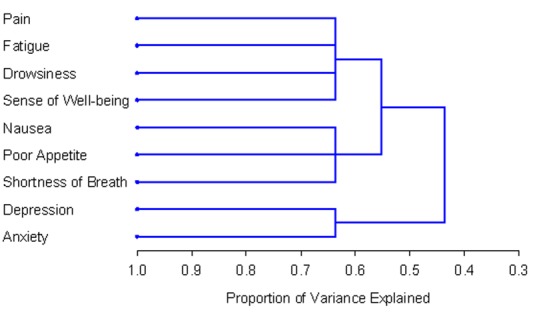
PROC TREE procedure generated dendrogram displaying three cluster solution and cluster hierarchy in 518 patients. Possible one cluster, two cluster and three cluster solutions explained 43.6%, 55.2% and 63.7% of the total variation, respectively.

**Figure 2:**
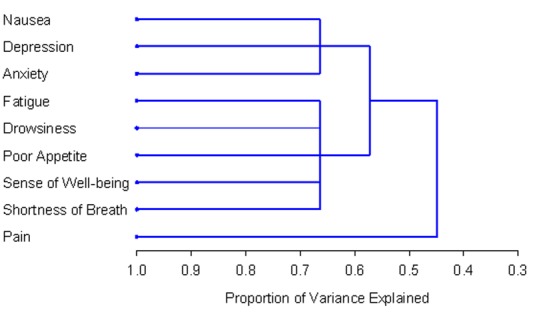
PROC TREE procedure generated dendrogram displaying two cluster solution and cluster hierarchy in 129 patients with non-zero record at baseline. Possible one and two cluster solutions explained 44.8% and 57.3% of the total variation, respectively.

The number and composition of symptom clusters identified from the total patient sample using HCA was identical to that when using PCA. The final three cluster solution in the total 518 patient sample explained 63.7% of the total variation. The tree diagram in Figure 1 is a visual representation of the three clusters identified. The clusters extracted from the non-zero subgroup differed from those identified using PCA by one symptom. Cluster 1 included depression, anxiety and nausea. Cluster 2 was composed of fatigue, drowsiness, appetite, shortness of breath and well-being. The final two cluster solution in the non-zero subgroup of 129 patients explained 57.3% of the total variation. Figure 2 shows the tree diagram illustrating the two symptom clusters identified. The symptom “pain” did not cluster with any of the other ESAS symptoms in the non-zero subgroup.

EFA derived two-factor (cluster) solutions upon analysis of both the original total cancer patient sample (n = 518; Table 3) and the non-zero subgroup (n = 129; Table 4). Cluster 1, derived from the total patient sample, included anxiety and depression. Cluster 2 was composed of the remaining seven ESAS symptoms. The Cronbach’s alpha for Clusters 1 and 2 was 0.82 and 0.81 respectively. In the non-zero subgroup, Cluster 1 was identical to Cluster 1 derived using HCA. Cluster 2 using EFA was very similar to the remaining cluster extracted using HCA, with the addition of the symptom “pain”. The Cronbach’s alpha for Clusters 1 and 2 in the non-zero subgroup were 0.76 and 0.80 respectively.

**Table 3:** Factor Loadings and Final Communality Determined Using Exploratory Factor Analysis in Total Patient Sample at Baseline (n = 518)

	Factor 1	Factor 2	Final communality
Drowsiness	0.67	0.20	0.49
Fatigue	0.66	0.23	0.49
Sense of Well-being	0.65	0.36	0.55
Poor Appetite	0.63	0.20	0.44
Pain	0.51	0.14	0.28
Nausea	0.49	0.26	0.31
Shortness of Breath	0.44	0.13	0.21
Depression	0.29	0.89	0.87
Anxiety	0.23	0.69	0.53
% of variance	82.5	17.5	
Cronbach’s alpha	0.82	0.81	
Eigenvalue	10.73	2.27	

Symptoms with bolded factor loadings were in the same cluster.

**Table 4:** Factors (Clusters) Determined Using Exploratory Factor Analysis in Non-zero Subgroup at Baseline (n = 129)

	Factor 1	Factor 2	Final communality
Drowsiness	0.76	0.18	0.61
Fatigue	0.72	0.34	0.64
Sense of Well-being	0.54	0.43	0.48
Shortness of Breath	0.53	0.30	0.37
Poor Appetite	0.43	0.31	0.28
Pain	0.16	0.14	0.04
Depression	0.25	0.91	0.89
Anxiety	0.36	0.72	0.64
Nausea	0.37	0.48	0.37
% of variance	85.8	14.2	
Cronbach’s alpha	0.76	0.80	
Eigenvalue	13.86	2.30	

Symptoms with bolded factor loadings were in the same cluster.

### Temporal pattern of symptom clusters in the non-zero subgroup

Among the 129 eligible patients at baseline, there were 64 patients at week 1, 65 patients at week 2, 62 patients at week 4, 56 patients at week 8, and 40 patients at week 12. The temporal pattern of symptom clusters was highly unstable in the non-zero subgroup regardless of the statistical method employed. The number and composition of symptom clusters identified using HCA and EFA varied significantly over time. PCA consistently identified two symptom clusters; however the composition of these clusters also varied considerably. Clusters derived using the three methods at each time point were rarely identical, the only exception being at one week where EFA and HCA identified the same clusters. The cluster findings over time are detailed in the Appendices (www.wjon.org).

While the temporal pattern of symptom clusters extracted using PCA, EFA and HCA were incongruent; the findings of all three methods are in agreement regarding the instability of clusters over time.

### Consistency of symptoms cluster findings using PCA, EFA and HCA

In general, there were significant variations in the quantity and composition of symptom clusters identified using the three statistical methods at each time point. Occasionally, cluster findings using PCA were identical to HCA, specifically at weeks 1, 2 and 12 in the total patient sample. As previously noted, in the non-zero subgroup analogous clusters were derived at one week using EFA and HCA. While the entire cluster composition varied over time, some symptoms within clusters consistently occurred in conjunction. Depression and anxiety always occurred together despite the time point or statistical method employed. Sense of well-being frequently occurred with these two symptoms as well. Also notable were fatigue and drowsiness, which often presented together at most time points regardless of the analytical method employed.

## Discussion

It is our understanding that this is the first study to identify symptom clusters over time using PCA, EFA and HCA in a subgroup of bone metastases patients reporting non-zero severity scores for all nine ESAS symptoms. The findings as assessed by three different statistical methods were compared with the clusters previously identified in the total patient sample at baseline.

In the present study, as in many previous studies statistically determining symptom clusters, clusters were extracted in accordance with their severity ratings. Many of these studies followed a short series of standard steps. First, patients indicated the presence and severity of each symptom they experienced through the use of an assessment tool. Next, statistical analysis, most often PCA, EFA or HCA, was applied to the symptom data compiled from all participating patients. Finally, symptoms were clustered based on their severities at each specific time point.

Previous studies tended to include all eligible consenting patients who provided complete symptom data, however this may distort the extraction of complete symptom clusters. A severity score of zero indicates the absence of a particular symptom, and the majority of the original patient sample population reported a zero score in regards to at least one ESAS symptom. This is a concern because the true definition of symptom clusters requires symptoms to co-occur, and therefore necessitating their presence, to be considered a part of the same cluster. Including patients who did not experience all symptoms evaluated in the assessment tool may be restrictive because such data does not provide a full overview of the interrelationships between all nine ESAS symptoms.

The present study revealed clear disparities between the symptom clusters identified in the non-zero subgroup and those extracted in the total sample population at baseline. As cluster incongruities are present at baseline between the two patient groups, we would reasonably expect disparities along the treatment trajectory as well. This indicates that the symptom experience of patients in the non-zero subgroup is not representative of those in the original sample population. Rather, the non-zero patients are a distinct subgroup that experiences unique symptom clusters. The observed disparities between the non-zero subgroup and total patient sample findings demonstrate the importance of screening patients based on their reported symptoms, especially when using short assessment tools such as the ESAS.

Our findings revealed that cluster results varied significantly when symptom and consequently patient inclusion criteria were slightly modified. This raises another concern of a required standard to determine when a symptom should be considered notable and included in symptom cluster analysis. Clusters in the present and many previous studies were identified based on the associations among symptoms which were determined by their numeric severity scores [5]. The ESAS, employed in this study, allows patients to rate nine symptoms on a scale of 0 to 10. Previous studies have categorized ESAS severity ratings into mild, moderate and severe categories based on the degree to which they interfered with the cancer patient’s function [13]. Ratings of 1-3 correspond to mild, 4-7 to moderate and 8-10 to severe symptoms. A symptom with a severity rating of 0 should rationally not be considered as it is absent in the patient. It may also be proposed that mild symptoms should be excluded as symptoms only become concerning to the patient when they noticeably affect the individual’s functioning or daily life. Further studies are warranted to assess any symptom cluster discrepancies in patients reporting exclusively moderate or severe symptoms.

The temporal pattern of symptom clusters may not be expected to remain stable over time, regardless of statistical method. Longitudinal data collected in palliative cancer patients using the ESAS showed that there was instability of symptoms and changing causal foundation of symptoms over time [14]. While certain clusters are consistent across studies, and are also clinically and statistically deemed as clusters (such as nausea-vomiting and anxiety-depression), other authors have stated that the stability of symptom clusters is unknown [15]. It may therefore be expected that the symptom clusters do not remain stable over time as patient’s disease progresses, particularly as patients in the non-zero subgroup reported in the current paper are likely of poorer health status [14, 15].

The use of different statistical methods to derive symptom clusters is a commonly discussed source of inconsistency. However, there are varying study results in the literature surrounding this notion. A few previous studies have noted similar, but not identical, cluster findings when different statistical methods are employed [16-18]. However, in our present study, clusters extracted using PCA, EFA and HCA were rarely similar. Although symptom clusters derived using HCA occasionally resembled those from PCA and EFA, no significantly consistent pattern of similarity was noted. The discrepancies in cluster findings between these three analytical methods indicate that the statistical method employed directly influences the clusters identified. Clinicians’ ability to apply findings to potentially improve treatment and symptom management is thus limited. There are certainly limitations associated with each method. For instance, factor analysis presumes that there is a stable common cause underlying the symptoms in any given factor. Clinically however, there may be different or changing causal factors underlying the relationships between symptoms over time [14, 19]. A universal analytical method agreed upon by both clinicians and statisticians will provide a basis upon which studies on symptom clusters for cancer patients can be compared.

The limitations of this study include the use of a brief assessment tool, such as the ESAS, which may lead to the extraction of oversimplified clusters. In addition, cancer patients encounter a wide array of symptoms, thus the nine-item ESAS may not effectively assess the comprehensive range of symptoms each patient is experiencing. This negatively impacts system clustering as the presence of additional clusters may be observed upon the addition of more specific symptoms. Also, the primary cancer each patient has been diagnosed with varies, thus different patient populations may experience a slightly different range of symptoms. Furthermore, since this study focused on the non-zero subgroup, this group of patients likely experienced more symptoms than the general bone metastases population. Therefore, the symptom cluster results may be more reflective of patients with poorer prognosis. The findings of our study should be interpreted with these considerations in mind.

Symptom cluster identification is a complex area of ongoing research in oncology. The present study identified two concerns for further investigation before the most relevant clusters can be extracted. The inclusion criteria for symptoms and therefore patients in symptom cluster analysis require further investigation and clarification. Additional studies employing various statistical methods are also necessary to determine the ideal statistical method to extract clusters in order to eliminate inconsistency. Increasing accuracy in symptom cluster studies is a promising area of research for developing new cancer treatment strategies, as proven by previous studies reporting the prognostic effect of multiple concurrent symptoms on functional status and quality of life [3, 20, 21].
